# Function-driven single-cell genomics uncovers cellulose-degrading bacteria from the rare biosphere

**DOI:** 10.1038/s41396-019-0557-y

**Published:** 2019-11-21

**Authors:** Devin F. R. Doud, Robert M. Bowers, Frederik Schulz, Markus De Raad, Kai Deng, Angela Tarver, Evan Glasgow, Kirk Vander Meulen, Brian Fox, Sam Deutsch, Yasuo Yoshikuni, Trent Northen, Brian P. Hedlund, Steven W. Singer, Natalia Ivanova, Tanja Woyke

**Affiliations:** 1 U.S. Department of Energy, Joint Genome Institute, Walnut Creek, CA 94598 USA; 2 Environmental Genomics and Systems Biology Division, Lawrence Berkeley National Laboratory, Berkeley, CA 94720 USA; 3 Joint BioEnergy Institute, Emeryville, CA 94608 USA; 4 Department of Biotechnology and Bioengineering, Sandia National Laboratories, Livermore, CA 94551 USA; 5 Department of Biochemistry, University of Wisconsin–Madison, Madison, WI 53706 USA; 6 Great Lakes Bioenergy Research Center, University of Wisconsin–Madison, Madison, WI 53706 USA; 7 Biological Systems and Engineering Division, Lawrence Berkeley National Laboratory, Berkeley, CA 94720 USA; 8 School of Life Sciences, University of Nevada, Las Vegas, Las Vegas, NV 89154 USA; 9 School of Natural Sciences, University of California Merced, Merced, CA 95343 USA

**Keywords:** Environmental microbiology, DNA sequencing, Biodiversity

## Abstract

Assigning a functional role to a microorganism has historically relied on cultivation of isolates or detection of environmental genome-based biomarkers using *a posteriori* knowledge of function. However, the emerging field of function-driven single-cell genomics aims to expand this paradigm by identifying and capturing individual microbes based on their in situ functions or traits. To identify and characterize yet uncultivated microbial taxa involved in cellulose degradation, we developed and benchmarked a function-driven single-cell screen, which we applied to a microbial community inhabiting the Great Boiling Spring (GBS) Geothermal Field, northwest Nevada. Our approach involved recruiting microbes to fluorescently labeled cellulose particles, and then isolating single microbe-bound particles via fluorescence-activated cell sorting. The microbial community profiles prior to sorting were determined via bulk sample 16S rRNA gene amplicon sequencing. The flow-sorted cellulose-bound microbes were subjected to whole genome amplification and shotgun sequencing, followed by phylogenetic placement. Next, putative cellulase genes were identified, expressed and tested for activity against derivatives of cellulose and xylose. Alongside typical cellulose degraders, including members of the Actinobacteria, Bacteroidetes, and Chloroflexi, we found divergent cellulases encoded in the genome of a recently described candidate phylum from the rare biosphere, Goldbacteria, and validated their cellulase activity. As this genome represents a species-level organism with novel and phylogenetically distinct cellulolytic activity, we propose the name *Candidatus* ‘Cellulosimonas argentiregionis’. We expect that this function-driven single-cell approach can be extended to a broad range of substrates, linking microbial taxonomy directly to in situ function.

## Introduction

The ‘rare’ biosphere comprises microbes that exist in low abundances. However, when conditions become favorable, the rare biosphere microbes can disproportionately affect ecosystem function. [1, 2]. Collectively, these microbes possess an exceptionally diverse set of enzymes [3], some of which may prove highly relevant to industrial processes, such as thermophilic cellulases for biofuels applications [4]. New opportunities to reveal these obscure microorganisms have arisen due to lower DNA sequencing costs that enable deep sequencing and advances in flow cytometry, imaging techniques, and high-throughput mass spectrometry. Combinations of these tools are beginning to elucidate functionally relevant organisms and associated enzymatic activities, which are critical to our understanding of both natural and engineered microbial systems.

Function-driven genomics provides a route to explore this dynamic functional diversity, and can be defined as any form of functional enrichment leading to the sequencing of genomes from either individual cells or consortia of cells [5]. Traditionally, stable isotope probing (SIP) has been combined with gas chromatography–mass spectrometry (GC–MS) to describe microbial diversity profiles based on traits of interest, such as the utilization of isotopically labeled substrates (typically ^13^C or ^15^N) [6]. While standard SIP was originally combined with phospholipid fatty acid profiling [7], DNA-SIP is now being rapidly adapted to high-throughput 16S amplicon [8] and metagenomic sequencing [7, 9]. Alternatively, new techniques and instrumentation are being developed including the application of ‘click’ chemistry to bioorthogonally label active cells in conjunction with fluorescence-activated cell sorting (FACS) [10] and the pairing of substrate-independent isotopic labeling (e.g., heavy water) with Raman imaging and isolation [11, 12]. Another highly relevant approach is to couple the fluorescent labeling of substrate molecules to FACS and shotgun sequencing. This technique has been previously used to identify active polysaccharide degraders in a coastal marine environment [13]. Importantly, this approach provides single-cell resolution, which is critical to understanding differences in gene content and niche differentiation among closely related individuals [14].

Here, we have employed a substrate labeling approach coupled with single-particle sorting and shotgun sequencing to identify putative cellulose degraders in a natural hot spring environment, followed by the expression and activity profiling of putative cellulose-degrading microbes. To benchmark the selectivity of our approach, we first tested our protocol using two cellulolytic bacteria, *Cytophaga hutchinsonii* and *Clostridium cellulolyticum*, by comparing their binding affinities to that of the noncellulolytic microorganism *Escherichia coli*. We then collected cellulose-degrading microbial consortia from a complex phototrophic microbial mat sample derived from a spring in the Great Boiling Spring (GBS) Geothermal Field in Gerlach, Nevada. The cellulose-binding assay was conducted on three separate incubations of this sample, providing one aerobic and two anaerobic samples, and shotgun sequencing was performed on all cellulose-bound particles. Amplicon sequencing was performed on the presorted communities to assess the level of enrichment. Finally, we identified putative cellulases from the genomes of the cellulose-bound microorganisms, expressed these enzymes and evaluated their activity using oxime derivatization of the sugar products and nanostructure-initiator mass spectrometry (NIMS) [15]. These data revealed cellulase activity across several taxonomic groups including Goldbacteria, a poorly understood candidate phylum found in the rare biosphere. Our findings emphasize the potential of a function-driven genomics approach to directly link function to the individual, highlighting its ability to enrich uncultivated microbes that would otherwise be too rare to capture with shotgun metagenomics of a bulk sample.

## Results and discussion

### Benchmarking of the fluorescently labeled cellulose approach

**Fig. 1:**
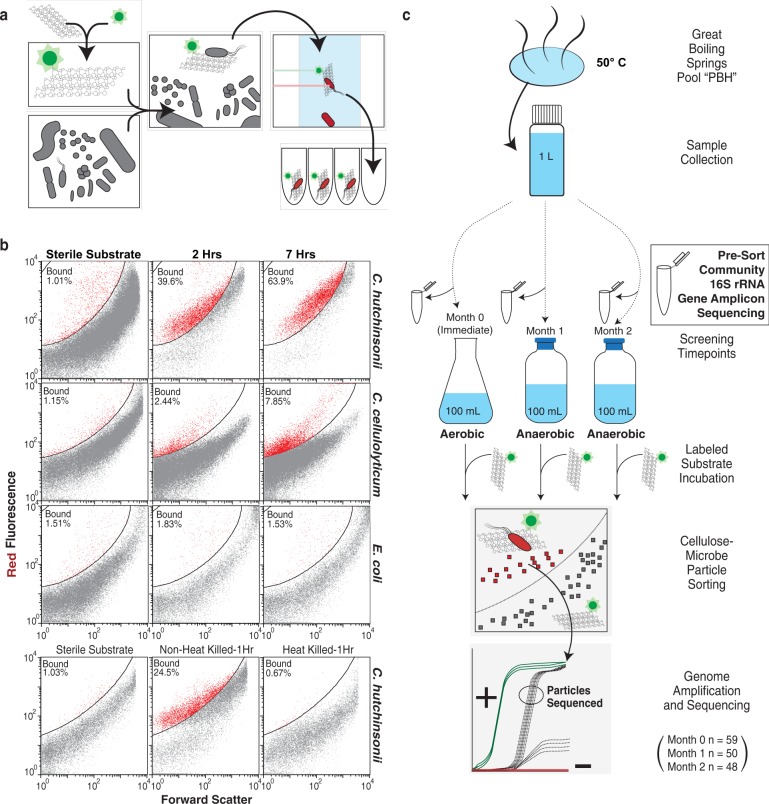
Function-driven single-cell genomics of cellulose-bound microbes. **a** Fluorescent substrate strategy used to identify microbes that colonize crystalline cellulose particles. The combination of a green fluorescent cellulose particle colonized by a red fluorescent DNA-stained microbe provides the signal to sort cellulose-adherent microbes from environmental samples. **b** Demonstration of flow cytometry gating strategy for identifying particles of labeled cellulose that become “bound” by microbes over time. Pure culture incubations of *C. hutchinsonii* (aerobic cellulose degrader), *C. cellulolyticum* (anaerobic cellulose degrader), and *E. coli* (noncellulose degrader) demonstrated specificity of binding to fluorescently labeled cellulose. Heat-killed *C. hutchinsonii* lost the ability to bind cellulose particles. **c** Experimental overview of function-driven sort and sequence data generated from Great Boiling Spring. Community composition of each bulk material (presort) was determined using 16S rRNA gene amplicon sequencing. Following incubation with labeled cellulose, cellulose-microbe particles were individually identified and sorted into 384-well plates. Whole genomes were amplified with multiple displacement amplification and the best amplification products based on real-time kinetics were sequenced to generate single-amplified genomes (SAGs). In total 59, 50, and 48 cellulose-microbe particles gave low crossing point (Cp) values (Supplementary Fig. 1) from time points 0, 1, and 2 months, respectively, and were sequenced yielding genomic information for a total survey of 157 cellulose-microbe particles

The model cellulose-degrading microbes *C. hutchinsonii* and *C. cellulolyticum* were used to develop, validate, and benchmark our cellulose-binding assay. These two organisms have been well studied for their ability to degrade cellulose through direct binding mechanisms under aerobic and anaerobic conditions, respectively [16–19]. *C. hutchinsonii* uses gliding motility and cell-associated (periplasmic and cell-surface) endoglucanases to degrade cellulose with minimal substrate loss, while *C. cellulolyticum* makes use of a multienzyme complex called the cellulosome. To demonstrate the efficacy of the approach, we labeled Arbocel ultrafine crystalline cellulose (UFC) with fluorescein and introduced the labeled substrate into pure cultures of these bacteria. Binding selectivity, based on the anticipated high affinity of some cellulose-degrading microbes for cellulose, was monitored using flow cytometry by first identifying the labeled cellulose particles, and then identifying the fraction that became bound by bacteria over time (Fig. 1a, b). SYTO red universal DNA stain was added to impart a red fluorescence signal characteristic of a bound microbe to the green fluorescent cellulose particle (Fig. 1a and Supplementary Fig. 1). In an exponentially growing culture of *C. hutchinsonii* we observed that ~40% of the cells were substrate bound after 2 h, and ~65% after 7 h of incubation, while for *C. cellulolyticum*, ~2.5% of the cells were bound to cellulose after 2 h and ~8% after 7 h (Fig. 1b). The noncellulolytic organism *E. coli* showed no binding in similar experiments and binding was undetectable in heat-killed *C. hutchinsonii* (Fig. 1b). Taken together, these data provided support for the efficacy of our approach and its utility for the targeted isolation of environmental bacteria that bind cellulose.

### Capturing and characterizing the cellulose-bound populations from Great Boiling Spring

Hot springs are attractive environments for studying cellulose decomposition because they continue to be a source of unexplored genetic diversity [20, 21], and the environmental conditions often mirror the pretreatment of lignocellulosic biomass before conversion to biofuels [22]. The hot spring sample used in the current study was collected from a pool in the GBS Geothermal Field. This geothermal system has been previously noted for the presence of cellulose-degrading thermophiles [23, 24]. A phototrophic mat/water sample was collected from the submerged Preston’s Brown Hole (PBH) pool and transported back to the lab at 50 °C to maintain in situ conditions, designated as “PBH” throughout this manuscript. After establishing that no native particles in the samples had fluorescent properties that overlapped with the signal from the fluorescein-labeled UFC, the labeled substrate was added to the hot spring sample and immediately screened at time 0 to establish the baseline properties of unbound cellulose (Supplementary Fig. 1). Following incubation, a bound population was detected on the labeled cellulose and was sorted in two stages—first, all bound cellulose-microbe particles were sorted in bulk into a single tube, then this bulk sample was resorted and individual cellulose-microbe particles were arrayed into 384-well plates (Supplementary Fig. 1). This two-stage approach ensured that all sequenced microbes could attach to cellulose strongly enough to withstand multiple rounds of sorting. The original PBH sample was sealed to exclude oxygen and left in the dark to incubate at 50 °C before repeating this screen at months 1 and 2 (i.e., addition of substrate, allowing the cells to bind, followed by sorting) (Fig. 1c). The months 1 and 2 samples are considered *anaerobic* throughout the manuscript based on the assumption that the community would rapidly consume all available oxygen. The rationale for including anaerobic incubations stems from our aim to capture both aerobic and anaerobic cellulose degraders from GBS, a spring which fluctuates between oxic and anoxic periods [25].

In total, 157 particles of cellulose that had been colonized by microbes with high binding affinity, hereafter referred to as cellulose-microbe particles, were genome sequenced (Fig. 1c). Particles were chosen for sequencing if their multiple displacement amplification (MDA) kinetics showed crossing points between 1.5 and 3 h that were well resolved from negative controls (Fig. 1c and Supplementary Fig. 1). This approach provides a taxonomy-agnostic screening strategy to sequence only single-amplified genomes (SAGs) or mini-metagenomes of high genome quality [26, 27] but may also bias the recovered population to cells that lysed most efficiently and yielded the highest levels of DNA, or particles bound by more than one cell. Of the 157 cellulose-microbe particles sequenced, 100 particles contained sequence from only one organism, while the remaining 57 particles had sequences from two (and in a few instances three) microbes (Supplementary Fig. 2). Genomes considered to represent the same species, as defined by average nucleotide identities greater than 95% [28], were co-assembled into CoSAGs for improved overall genome completeness of the resulting population genomes (Supplementary Table 1).

**Fig. 2:**
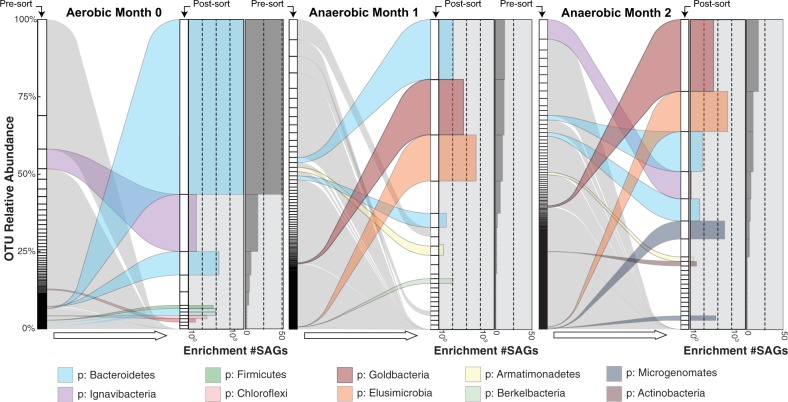
Sorting enrichment of SAGs recovered from sample PBH at time points 0, 1, and 2 months. Relative abundances of individual operational taxonomic units (OTUs) in bulk sample before sorting determined by 16S rRNA gene sequencing (iTags) and recovered SAGs after sorting, amplification and sequencing are plotted. Original sample OTUs that match to a recovered SAG that were enriched during the sort are marked with a colored alluvium indicating taxonomy (phylum) that links the pre- and post-sort relative abundances. SAGs with no assembled 16S rRNA genes do not have colored alluvium linking them to their original abundance. The enrichment plot quantifies the relative increase of that OTU following sorting and the total number of recovered SAGs

The enrichment of specific microbial taxa using our *bait* and *hook* approach (e.g., cellulose particles are the *bait*, while the FACS sorting acts as the *hook*) is illustrated in Fig. 2, where the relative taxa abundances of the presorted/bulk communities obtained via 16S rRNA gene amplicon sequencing are shown next to the cellulose-bound communities. The cellulose-bound microbes represented a minute fraction of the overall diversity observed in the bulk community. While all SAG species collected with our approach were present in the bulk communities, the enriched taxa were found at extremely low abundances. The phylum-level clades with significant enrichment included the Bacteroidetes, Ignavibacteria, Chloroflexi, Goldbacteria, Elusimicrobia, and the Microgenomates (Fig. 2). The most highly enriched taxon belonged to the Bacteroidetes phylum (GenomeID: Bacteroidetes GBS-CoSAG_01, Supplementary Table 1). This taxon represented 52 out of 92 of the SAGs recovered from the aerobic month 0 sample (Fig. 2, Supplementary Fig. 2 and Table 1), though it was only present at a 0.006% relative abundance in the bulk community, a total enrichment of 9150 ×. Based on its 16S rRNA gene sequence, Bacteroidetes GBS-CoSAG_01 is a divergent member of the Bacteroidetes and places within the Bacteroidetes VC2.1 Bac22 order comprised of environmental 16S rRNA genes sequences. Under anaerobic conditions, Bacteroidetes GBS-CoSAG_01 was not recovered in the assay, and its absence in the community at the anaerobic month 1 time point indicates it was displaced under anaerobiosis. The largest enrichment under anaerobic conditions occurred for a member of the Elusimicrobia phylum, with an average enrichment of 500x across the two time points (GenomeID: *Elusimicrobia* GBS-CoSAG_05, Table 1, Supplementary Table 1). Approximately 15% of SAGs recovered from anaerobic samples belonged to this group, though it was detected at only 0.02% in the bulk community, for a total enrichment of ~500 × (Fig. 2 and Supplementary Fig. 2). This member was present in the initial sample at month 0, but represented only 0.01% of the community, indicating its affinity to cellulose, and its ability to proliferate in an anaerobic environment.

**Table 1:** Assembly statistics for the most enriched members of our function-driven screen alongside the Goldbacteria metagenome-assembled genomes collected from IMG

Source	IMG Taxon ID	Organism name	Est. completeness, % (CheckM)	Est. contamination, % (CheckM)	Assembly size, Mb (IMG)	% GC (IMG)	Number of contigs (IMG)	Contig N50, kb (CheckM)	Gene Count (IMG)	Coding density, % (IMG)
Coassembly	2795386099	Bacteroidetes bacterium GBS-CoSAG_01	98.39	0.81	2.66	36.9	128	38.4	2447	92.6
Coassembly	2795386067	Ignavibacteriales bacterium GBS-CoSAG_02	94.57	2.46	3.37	32.4	200	39.3	2990	90.3
Coassembly	2795386064	Sphingobacteriales bacterium GBS-CoSAG_03	96.11	0.65	3.04	30.8	128	53.2	2717	90.4
Coassembly	2795386070	Candidatus Cellulosimonas argentiregionis GBS-CoSAG_04	92.7	2.25	2.74	29.8	278	20	2615	91
Coassembly	2795386065	Elusimicrobia bacterium GBS-CoSAG_05	80.26	0	1.53	32.3	149	18.8	1523	91.9
Coassembly	2795386068	Bacteroidales bacterium GBS-CoSAG_08	92.89	6.92	3.77	44.9	477	15.9	3519	90.1
IMG MAG	2795386097	Candidatus Goldbacterium OP01	76.46	0	1.58	30	212	9.2	1542	88.4
IMG MAG	2795386098	Candidatus Goldbacterium UKC048	73.03	1.12	1.89	43.5	99	40.3	1692	92.1
IMG MAG	2795386096	Candidatus Goldbacterium DC9	98.88	8.99	2.65	29.9	259	14.9	2459	89.2
IMG MAG	2784132058	Candidatus Goldbacterium DG074	68.66	1.75	1.25	45.6	240	5.6	1297	96.4
IMG MAG	2784132059	Candidatus Goldbacterium DG078	94.38	1.23	1.99	44.8	210	12.4	2026	95.2

**Fig. 3:**
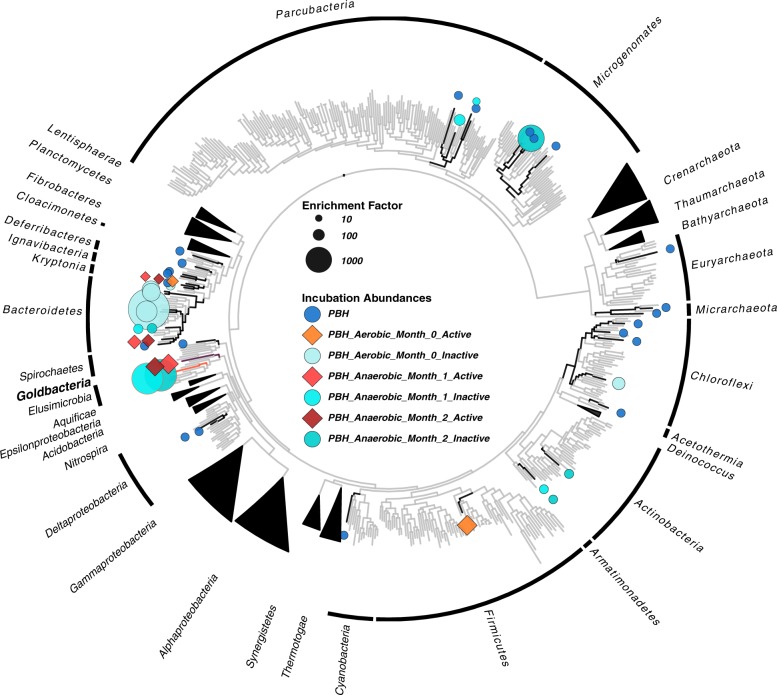
Phylogenetic placement of all enriched SAGs and Co-assembled SAGs (CoSAGs) in a whole genome tree based on concatenated alignments of 56 conserved marker genes. Cellulose-captured SAGs are represented by black branches, while gray branches represent a dereplicated version of the IMG reference database. References include roughly 600 genomes, reduced from ~60,000 using RNA polymerase beta-subunit clustering at 65% similarity. Symbols refer to the presence of at least one active cellulase (diamonds) or no active cellulases (circle) determined via the NIMS-Oxime assay (Supplementary Fig. 8). The bubbles note the enrichment factor which corresponds to the same taxa shown in Fig. 2. Only SAGs of high taxonomic confidence whose placement agreed with its CheckM and/or 16S rRNA gene-based taxonomy were included. The candidate phylum Goldbacteria representative *Candidatus* ‘Cellulosimonas argentiregionis’ CoSAG, and the highly enriched co-sorted bacterium *Elusimicrobia* GBS-CoSAG_05 are displayed with purple and orange branches, respectively

Placing only medium- and high-quality SAG and CoSAG genomes of at least 50% estimated genome completeness (Table 1) [29] into a phylogenomic context showed that many of the recovered PBH genomes are closely related to microbes previously described as cellulose degraders (Fig. 3), including the Bacteroidetes, Ignavibacteria/Kryptonia, Firmicutes, Chloroflexi, Actinobacteria, and Spirochaetes (Table 1) [30–36]. From these genomes, we predicted the potential mechanisms enabling cellulose adhesion. This includes proteins with domains or combinations of domains described to be involved in cellulose degradation [37] such as glycoside hydrolases (GH) of which cellulases are a specific subclass, carbohydrate-binding modules (CBMs) and other cellulose adhesion strategies [38, 39] (Supplementary Fig. 3). Though the presence of a domain alone is insufficient to attribute cellulolytic function to these candidate enzymes, many validated cellulases demonstrate similar architecture with the presence of specific cellulose-binding modules attached to glycoside hydrolases known for activity on cellulose [40].

Based on a 16S rRNA gene-based phylogeny, the most novel of the recovered microorganisms falls within the proposed bacterial candidate phylum FCPU426, which contains a 16S rRNA gene sequence from the recently described candidate phylum Goldbacteria [41] (Supplementary Fig. 4). The pairwise AAI between our CoSAG and the published Goldbacteria metagenome-assembled genome (MAG) (2.89 Mb and 99% estimated (est.) completeness) is 54%, placing our CoSAG as a new genus level member of the Goldbacteria phylum. For this organism we propose the genus species name, *Candidatus* ‘*Cellulosimonas argentiregionis*’ (GBS; the type material is IMG Genome ID = 2795386070). The etymology behind this name is as follows: Cellulo refers to the substrate (cellulose) that single cells of this microbe were recovered on, and argentii refers to its recovery from Nevada, the silver state (and its emergence as the second genome in the proposed Goldbacteria phylum). *Ca*. ‘Cellulosimonas argentiregionis’ was enriched 60x above its environmental abundance in our assay and yielded the most SAGs from the anaerobic samples (*n* = 28). Coassembly of these SAGs produced a high-quality Co-SAG (Co-assembled-SAG) from the anaerobic incubation (Table 1 and Fig. 2), and contained the largest suite of genes coding for potentially cellulolytic enzymes (Supplementary Fig. 5).

### Characterization of a cellulose-degrading candidate phylum retrieved from the rare biosphere

**Fig. 4:**
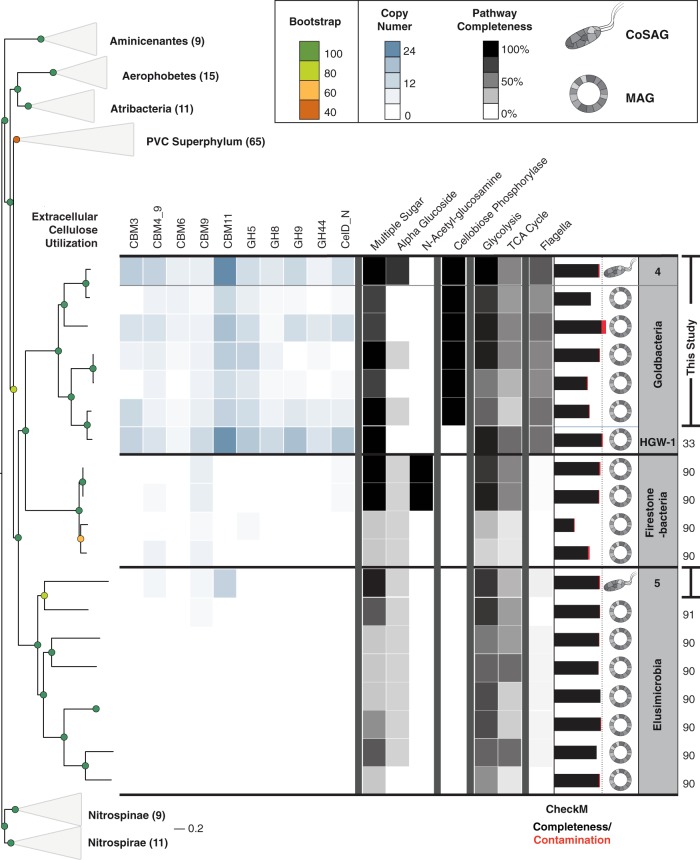
Characterization of our Goldbacteria genome (GBS-CoSAG_04) based on its placement in a single-copy marker gene tree together with its nearest neighbors: Firestonebacteria and Elusimicrobia, the additional MAGs extracted from published metagenomes ( Supplementary Table 2) and the previously published MAG, HGW-1 [41]. The heat map displays the families (Pfams) and KEGG Orthology (KO) terms associated with degradation, internalization, and central metabolism of cellulose as a substrate. Of note is the clear absence of cellulose degrading and flagellar genes in the Goldbacteria sibling clades, Firestonebacteria and Elusimicrobia. IMG metagenomes where MAGs were recovered are described in Supplementary Table 2. Brackets at far right indicate genomes derived from his study while numbers correspond to the following genomes/metagenomes available in the public databases [41, 86, 87]

Genomic data from the candidate phylum Goldbacteria has been recovered only once prior to this study as a MAG from the deep subsurface (2.86 Mb and 99% est. completeness), noted for its large complement of putative cellulases [41]. The co-assembled genome sequence of *Ca*. ‘*C. argentiregionis*’ has a size of 2.74 Mb (est. completeness of 92.7%, est. contamination of 2.25%) and based on the presence/absence of KEGG pathway information encodes the potential to degrade cellulose to glucose, which is available for anaerobic fermentation. To validate Goldbacteria as a distinct major lineage in the bacterial tree of life, along with our co-assembled single-cell genome of *Ca*. ‘C. argentiregionis’ (GBS-CoSAG_04), we extracted additional MAGs from ~6000 publicly available assembled metagenomes in JGI’s Integrated Microbial Genomes and Microbiomes (IMG/M) system [42] to help resolve the placement and conserved features inherent to the candidate phylum Goldbacteria (Fig. 4, Table 1 and Supplementary Table 2).

**Fig. 5:**
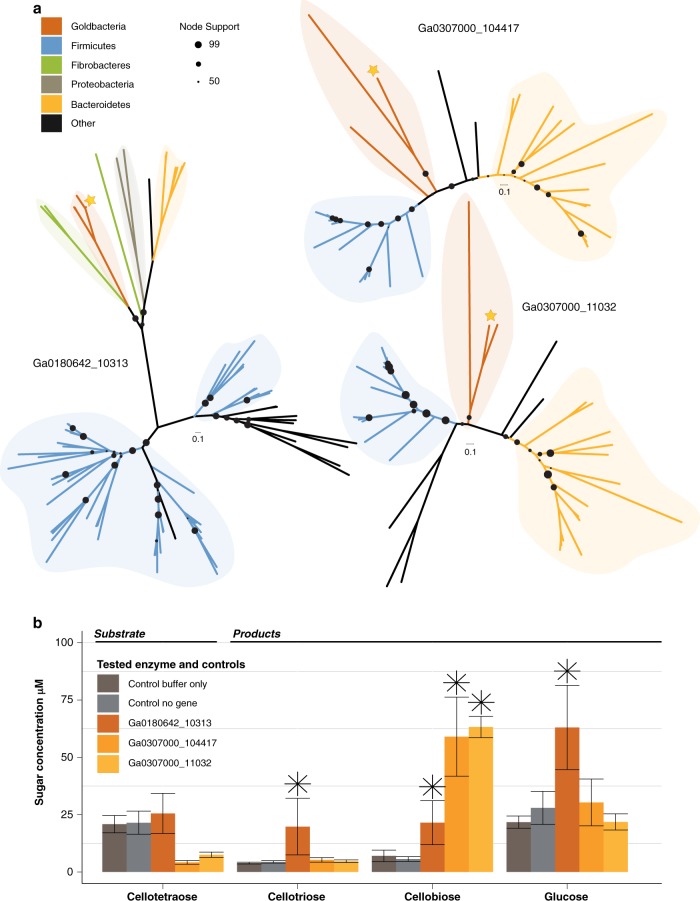
**a** Protein trees displaying heterologously expressed Goldbacteria cellulases alongside their nearest phylogenetic neighbors based on BLAST searches against the NCBI nonredundant database. Goldbacteria cellulases are shown with red branches, and the yellow star specifies the gene from *Ca. ‘*Cellulosimonas argentiregionis’ GBS-CoSAG_04 (IMG Genome ID 2795386070) corresponding to the IMG gene IDs adjacent each tree. **b** Activity measurements of the same three proteins. The bar chart displays the initial concentration of Cellotetraose alongside the three main enzymatic products of the tested cellulases. For an enzyme to be considered positive (denoted by the asterisk), the product concentration had to be 2X above the mean control concentration

Though a range of CBMs known to be involved in cellulose binding were encoded in the *Ca*. ‘*C. argentiregionis*’ genome, such as CBMs 3, 4, 6, and 9, CBM11 was the most prominent with 24 copies. Similarly, a high copy number of putative cellulases containing GH families 5, 8, 9, and 44 were also present within most of the Goldbacteria genomes indicating that Goldbacteria harbors a suite of carbohydrate-active enzymes for substrate binding and deconstruction (Supplementary Fig. 5). As expected, complete downstream pathways for utilization of cellulose hydrolysis products, including cellobiose phosphorylase, glycolysis, and a partial TCA cycle were also conserved. Interestingly, the phylogenetically most closely related phyla, the Firestonebacteria and Elusimicrobia (of which the PBH sample also contained), largely lacked this putative cellulase machinery (Fig. 4) and have not been previously predicted to be involved in cellulose degradation as a primary lifestyle [43–45]. However, we should also note that our Elusimicrobia CoSAG (GBS-CoSAG_05) did contain a minimal set of CBMs, indicating that Elusimicrobia may be able to bind, but not degrade cellulose (Fig. 4 and Supplementary Fig. 5). Moreover, single protein phylogenies showed that the predicted cellulases of *Ca*. ‘C. argentiregionis’ (GBS-CoSAG_04) are highly similar to the predicted cellulases within the Hernsdorf MAG [41] and are related to some more well-characterized cellulose-degrading bacterial cellulases, including those found in the Bacteroidetes and Firmicutes. In one case, a predicted Goldbacteria cellulase may have arisen through horizontal gene transfer (Fig. 5a).

Expanding the genomic representation and experimental validation of gene function within the candidate phylum Goldbacteria is important for the authentication of the FCPU426 16S rRNA gene cluster, a candidate phylum clade previously composed of only environmental 16S rRNA gene library sequences. The *Ca*. ‘C. argentiregionis’ 16S rRNA gene sequence robustly grouped within this candidate phylum, with full-length sequence identities ranging from 79 to 88% and the most closely related sequence identified in a wetland rhizosphere (Supplementary Fig. 4). This placement suggests that the 16S rRNA sequences previously belonging to the FCPU426 cluster are indeed members of the Goldbacteria phylum.

In order to assess the abundance and distribution of the Goldbacteria, 16S rRNA gene sequences similar to *Ca*. ‘*C. argentiregionis*’ were recovered from IMG/M, Jan. 2018 [42]. Goldbacteria-like sequences were only detected in 46 of 6413 assembled metagenomes (Supplementary Fig. 6). The environmental distribution of these 16S rRNA gene sequences included a wide range of habitats and growth temperatures, including not only hot springs and the terrestrial subsurface, but also freshwater, soil, marine, and engineered systems such as anaerobic digesters. Furthermore, in a majority of these locations, the Goldbacteria-like sequences had low estimated relative abundances (0.1–1%) (Supplementary Fig. 6), suggesting that members of Goldbacteria are members of the GBS rare biosphere. Mapping reads of all Goldbacteria-containing publicly available metagenomes to our representative SAG and MAG genomes supported this low abundance hypothesis: only a small percentage of reads (0.6–1.2%), with low median read coverage (13× to 32×) mapped to each reference from their source metagenomes (Supplementary Fig. 7 and Supplementary Table 2). These findings are in line with previous abundance estimates. A 2012 16S rRNA gene amplicon survey detected the first sequence belonging to the FCPU426 candidate phylum in the Zodletone Spring [46]. Targeted qPCR of this sample determined that this member was part of the rare biosphere, present at only a 0.4 ± 0.06% relative abundance in the community [46]. This abundance value is comparable with our iTag-based detection of 0.3% for *Ca. ‘C. argentiregionis*’ under anaerobic conditions (an increase from the 0.013% original abundance in the unincubated sample).

### Experimental validation of candidate cellulases

A total of 70 candidate cellulases (excluding CBMs) from all taxa with putative cellulases were further characterized by expression in a cell-free translation system and oxime-NIMS detection of reducing sugar release [47, 48]. Based on the conditions of the assay, 16 enzymes were catalytically active against derivatives of lignocellulose (12 enzymes showing activity against cellotetraose and 6 against xylotetraose). Overall, members of the Bacteroidetes, Ignavibacteria/Kryptonia, Chloroflexi, Firmicutes, Deferribacteres, and Goldbacteria had at least one active enzyme. Interestingly, the Bacteroidetes genome harboring the highest number of active enzymes using our assay (*n* = 5; Bacteroidetes GBS-CoSAG_08), Supplementary Fig. 8) was not the Bacteroidetes with the highest enrichment (Bacteroidetes GBS-CoSAG_01). Instead, it was a Bacteroidetes genome that was enriched from the anaerobic time points (Figs. 2 and 3), suggestive of specialization for cellulose degradation within this clade under anaerobic conditions at GBS.

With the exception of Goldbacteria, all taxa recovered using our *bait* and *hook* approach belong to phyla previously observed to contain active cellulases (Fig. 3). Since the Goldbacteria genome harbors the highest number of cellulases identified (Supplementary Fig. 5), we focus the remainder of our discussion on the enzymes observed from this candidate phylum. Of the 70 candidate cellulases observed in PBH genomes, 21 belonged to *Ca*. ‘C. argentiregionis’. All of these contain GH domains previously implicated in cellulose deconstruction, and three *Ca*. ‘*C. argentiregionis*’ enzymes (Ga0307000_104417, Ga0307000_11032, and Ga0180642_10313) liberated cellobiose (with Ga0180642_10313 also liberating glucose) from the cellulosic substrate (Fig. 5b and Supplementary Fig. 8). These three enzymes belong to the GH 9 family of endoglucanases, which is a highly diverse family of cellulases [30, 49]. Ga0307000_104417 contains a cellulase N-terminal immunoglobulin-like domain, while Ga0180642_10313 and Ga0307000_11032 contain GH 9 domains. When placed into a phylogenetic context (Fig. 5a), these three enzymes formed monophyletic groups together with cellulases from the deep subsurface Goldbacteria MAG [41]. Altogether, the phylogenetic placement of the Goldbacteria cellulases recovered from PBH demonstrates a potentially unique evolutionary history and provides additional support that *Ca*. ‘*C. argentiregionis*’ is a novel microbe with a unique assemblage of cellulose-degrading enzymes. Cellulases encoded by two genes, Ga0307000_104417 and Ga0307000_11032, clustered together at the base of cellulase genes from the Firmicutes, suggesting shared ancestry. However, GH 9 Ga0180642_10313, grouped within cellulase-annotated proteins from the Fibrobacteres, suggesting this gene may have been more recently acquired in the Goldbacteria through horizontal gene transfer from the Fibrobacteres (Fig. 5a). While the GH 9 endoglucanase family has not previously been defined as a horizontally acquired gene, a number of carbohydrate-active enzymes have been observed as horizontally transferred genes between anaerobic gut fungi and bacteria [50].

### Co-sorted cells

Through our function-driven small particle sequencing approach, we recovered a targeted snapshot of microbial activities and interactions occurring at the microscale on a single particle of cellulose. The resolution of microbial spatial arrangements preserved within the sequence data provides insights into spatiotemporal interactions of individual microbes within this environment. Elusimicrobia GBS-CoSAG_05 (Fig. 2. months 1 and 2 Elusimicrobia, Table 1) is another low abundance member of the PBH community (<1%), yet Elusimicrobia GBS-CoSAG_05 cells were enriched over 500x during the sort, demonstrating a strong affinity for cellulose. Elusimicrobia, formerly known as Termite Group 1 [51], have been previously observed as symbionts in the guts of termites [52], cockroaches, and ruminants [53]. Elusimicrobia appear to be strictly anaerobic, growing on glucose while producing lactate, acetate, hydrogen, and CO_2_ via fermentation [45], identified as gut symbionts, and more recently from aquifer sediment samples [54], and now in our GBS sample. Interestingly, nearly 80% (15/19) of the observed Elusimicrobia SAGs co-occur with *Ca*. ‘*C. argentiregionis*’ on the same sorted cellulose particle (Supplementary Fig. 2), with the other four occurrences having no partner. These results might suggest that the co-occurrence of *Ca*. ‘*C. argentiregionis*’ and *Elusimicrobia* GBS-CoSAG_05 on the same particle is either the result of an association where *Elusimicrobia* GBS-CoSAG_05 relies on *Ca*. ‘C. argentiregionis’ for the liberation of substrate, or that these microbes compete for substrate as both individuals were, at times, sorted independently. Either way, it is intriguing to note that while these two microbes make up less than 0.5% of the total community, their prominence as the most abundant cellulose colonizers suggests an association with cellulose.

## Concluding remarks

By deploying a targeted screen into this hot spring community, we recovered a snapshot of relevant microbes that were the initial recruits to crystalline cellulose. Though many of these microbes were present at <1% relative abundance within this environment, the enrichment and sequence recovery from these cellulose-adherent microbes demonstrated the strength of the function-driven single-cell genomics approach. Together, this suggests that the sorting assay was highly specific in recovering diverse members from the functional niche that rapidly responded to the cellulose substrate. This approach provides a sensitive, cultivation-independent strategy to answer the specific ecological question of by whom, and how, cellulose is degraded in this environment. Of the 40 novel clades recovered in this study, a majority represent uncharacterized lineages, many with full-length 16S rRNA gene sequence identities in the low to mid 80% range to their nearest relatives. The recovery of a large number of cells from the rare biosphere population of *Candidatus* ‘Cellulosimonas argentiregionis’ allowed us to reconstruct a high-quality draft co-assembled genome to gain insight into the new Goldbacteria phylum and uncover carbohydrate-active enzymes likely responsible for Goldbacteria’s growth on cellulose. Through strong enrichment of a new phylum, the bait-and-hook approach described here provides a new, single organism-level way to carry out bioprospecting. Finally, experimental validation of the cellulases encoded in the recovered genome demonstrated that in addition to the opportunity to recover novel taxa, function-driven single-cell genomics offers a targeted approach for identifying enzymatic activities of interest arising from seldom-cultured microbes. By applying this approach to other substrates, and by combining single-cell genomics with advancing metabolite detection methods, the scope of in situ functions that can be characterized in uncultivated environmental microbial populations will continue to expand.

## Methods

### Substrate preparation

The microbial substrate used for both benchmarking and in the GBS field sample experiments was Arbocel Untrafile Cellulose provided by Marc Mohring at J. Rettenmaier & Söhne, labeled with fluorescein. Microcrystalline cellulose was selected as substrate based on its small particle size (~8 μm) which limits the number of cells that can attach to an individual particle. The synthesis was performed by mixing 1 g of Arbocel Ultrafine Cellulose (J. Rettenmaier & Söhne, Rosenberg, DE.), with 60 mg of 5-(4,6-Dichlorotriazinyl) aminofluorescein (5-DTAF) (Sigma Aldrich, St. Louis, MO.) in 10 mL of 0.1 M NaOH for 1 h. Cellulose was then washed by three repeated cycles of collection on a 0.1 μm filter, resuspension, and vortexing in 10 mL of sterile MilliQ water to remove unreacted 5-DTAF. The final solution was resuspended in a small volume of sterile phosphate buffered saline (PBS) and stored as a slurry.

### Cultivation

Two known cellulose degraders, an aerobic bacterium, *C. hutchinsonii*, and an anaerobic bacterium, *C. cellulolyticum*, (ATCC, Manassas, VA.) were used as positive controls in our cellulose-binding experiments, and *E. coli* BL21 as negative control (noncellulose degrader). *C. hutchinsonii* was cultivated using ATCC medium 1160 with Arbocel UFC substituted for filter paper, and *C. cellulolyticum* was cultivated in DSMZ 530 media with Arbocel UFC (J. Rettenmaier & Söhne, Rosenberg, DE.) substituted for cellobiose. *E. coli* BL21 was cultivated in modified ATCC PYG media (glucose was replaced with Arbocel UFC) (Supplementary Table 3).

### Flow cytometry

All experiments were performed using a Becton Dickinson Influx flow cytometer (Becton Dickinson Co., Franklin Lakes, NJ) with a 100 μm nozzle. Prior to operation, fluidic lines were sterilized by flowing through 1 L of a 10% bleach solution for 2 h. Sheath fluid consisted of 1X PBS made from a 10X OmniPur® PBS stock (VWR, Radnor, VA) in 18.2 MΩ milliQ water (EMD Millipore, Billerica, MA) and was treated by overnight UV irradiation [55]. During operation, the sheath tank was pressurized at 8.5 PSI and the waste tank vacuum was applied at 10 PSI. Following stabilization of the fluidics and establishment of a stable stream breakoff, blue (488 nm) and red (642 nm) lasers were aligned using Sphero^TM^ Rainbow Fluorescent Particle beads (Becton Dickinson and Co., Franklin Lakes, NJ). Photomultiplier voltages were adjusted to center on the 1–5 μm range of cellulose particles.

### Great Boiling Spring sample collection, lab incubations, and flow cytometry

Geothermal spring samples were collected from a pool (PBH) near the north end of the GBS Geothermal Field, outside of Gerlach, NV (40.663, −119.367). Biofilm and water were collected from a submerged root in the GBS, PBH pool, using a sterile 1 L polypropylene copolymer Heavy-Duty Nalgene bottle (VWR, Radnor, PA.). Accessory data, including temperature and pH of the spring, were recorded at time of sampling (Supplementary Table 3). While dissolved O_2_ was not measured during sampling, previous measurements have been recorded by Murphy et al. [25]. Immediately after sampling, the container was sealed and put into a 50 °C preheated portable incubator. The sample was then transported back to the lab by car (~5 h).

Upon arrival of the sample at the lab, a 100 mL aliquot of the sample slurry was added into a sterile 250 mL Erlenmeyer flask. The sample (month 0) was screened by flow cytometry to determine initial bacterial cell counts by diluting 100 μL of this sample in 890 μL sterile PBS and staining with 10 μL of a cocktail of 50 μM SYTO 17/59/61 DNA stains (Supplementary Fig. 9). Fluorescently labeled microcrystalline cellulose (Arbocel UFC) was screened with the 488 nm laser and visualized for green fluorescence (531 ± 20 nm) and forward scatter. This resolved population was identified as the labeled substrate (Supplementary Fig. 1). The GBS/PBH sample was screened to ensure no preexisting populations overlapped with the gate drawn for the labeled substrate. Using counts from the GBS/PBH sample, the labeled cellulose was then introduced to a target concentration of ~1% of all events. Screening the substrate-amended culture, fluorescently labeled cellulose particles were first identified as a continuous population with an exponential 531 nm fluorescence signal when plotted vs. forward scatter (Supplementary Fig. 1). A time series of the incubation, starting at time 0 h (initial addition of the labeled substrate) monitored binding affinities of the culture with the labeled cellulose. Detection of binding was achieved first gating on the green fluorescent signal of the cellulose, then revisualized for their red fluorescence (670 ± 20 nm) from excitation with the 642 nm laser vs. forward scatter (Supplementary Fig. 1). Once at least 5% of cellulose particles were measured as bound, the cellulose-microbe population was sorted into a single sterile cytometry tube. After collecting ~250,000 of these events, the cellulose-microbe particles were resorted from the cytometry tube and individually index sorted into UV-treated 384-well plates. Positive (100 bound particles/well) and negative (1 sterile sheath, 1 unbound fluorescent cellulose particle/well) controls were included as dedicated columns in each sorted plate. Following sorting, plates were immediately sealed, centrifuged at 1000 *g* for 1 min, and frozen at −80 °C.

Anerobic incubations (months 1 and 2) were set up using an anerobic chamber under 80% N_2_, 15% CO_2_, and 5% H_2_. All manipulations of the cultures were performed inside the chamber and cultures kept sealed with butyl stoppers and using a crimp top during incubation at 50 °C for months 1 and 2, respectively. Anaerobic incubations were screened by flow cytometry as outlined for the month 0 sample.

### 16S rRNA gene amplicon sequencing

For universal amplification of the V4 region of the 16S rRNA gene (V4 iTags), we used forward primer 515F (5′-GTGCCAGCMGCCGCGGTAA-3′) and reverse primer 806R (5′-GGACTACHVGGGTTCTAAT-3′) containing a variable 12 bp barcode sequence [56]. Pooled amplicons were purified with the Agencourt AMPure XP purification system (Beckman Coulter, Brea, CA, USA) and analyzed with an Agilent bioanalyzer 2100 (Agilent Technologies, Palo Alto, CA, USA) to confirm appropriate amplicon size. iTag sequencing was performed according to JGI’s standard procedures: iTag amplicons were diluted to 10 nM, quantified by quantitative PCR and sequenced on the Illumina MiSeq platform (reagent kit v.3; Illumina Inc., Carlsbad, CA, USA). iTag sequences were analyzed using the JGI iTagger analysis pipeline [57].

### Single-cell genome sequencing

All single-cell MDA products were obtained as previously described [55]. Briefly, sorted cells in 384-well plates were subjected to lysis to liberate DNA from sorted cells for amplification. Lysis and MDA reagents were quality controlled for purity by including one row of negative controls lacking sorted cells (“no sort” control) in each microtiter plate and monitoring amplification kinetics. Using an Echo® 550 liquid handling system (Labcyte, Sunnyvale, CA), lysozyme, KOH, and a neutralization buffer were subsequently transferred into individual wells for lysis [58]. MDA was then performed using the RepliG kit (Qiagen, Hilden, DE) according to manufacturer’s directions, but adjusted for 1.2 μL reactions. The MDA reaction was incubated at 31 °C for 6 h before the amplification was stopped and the polymerase heat-killed. MDA kinetics were monitored in real time using SYTO 13 green to determine successfully amplified wells. One hundred and fifty-seven wells from the three different time points (months 0, 1, 2) with the best MDA kinetics based on lowest crossing point values were chosen for shotgun sequencing. “No sort” control wells did not yield any detectable DNA amplification. Indexed Nextera libraries (Nextera XT kit, Illumina) were generated, which were then pooled and sequenced according to standard JGI procedure using the Illumina Nextseq platform (https://www.illumina.com/systems/sequencing-platforms/nextseq.html).

### Genome assembly and annotation

Sequences from single cellulose-microbe particles were assembled using SPAdes (version 3.11.1) [59] with the single-cell flag and using 57, 92, and 127 kmers. Following initial assemblies of individual cellulose-microbe particles, all fastq files were profiled against all single-particle assemblies using Anvi’o [60] to visualize mapped reads and identify SAGs of the same organism on different particles (i.e. MAGs or *metagenome-assembled genomes* from particles with multiple cell types). Recovery of the same species across all genomes derived from different particles was calculated using an average nucleotide identity species threshold of 95% [61] or greater across using pyani.py (https://github.com/widdowquinn.pyani). For microbial species recovered multiple times, co-assemblies of these fastq files were created to generate more complete genomes using SPAdes with the trusted contigs flag. All genomes were curated based on tetranucleotide frequency of contigs using MetaBAT [62] and checked for quality with CheckM [63]. Gene calling was performed with Prodigal [64]. Pfams of interest were downloaded from the Pfam portal at EMBL-EBI [65]. Genes of interest were identified using hmmsearch from the hmmer package [66]. Pathways for metabolism were identified as KEGG references [67].

### Phylogenetic placement of putative cellulose degraders and associated bacteria

A set of 56 universal single-copy marker proteins [68, 69] was used to build phylogenetic trees for bacteria and archaea based on all available publicly accessible microbial genomes in IMG (download October 27, 2017) (Fig. 3). Marker proteins were identified with hmmsearch (version 3.1b2, hmmer.org) using a specific hidden-markov model for each of the markers. Genomes lacking a substantial proportion of marker proteins (more than 28), or which had additional copies of more than three single-copy markers, were removed from the dataset. To reduce redundancy, DNA directed RNA polymerase beta subunit 160 kD (COG0086) was identified and clustered with cd-hit [70] at 65% sequence similarity, resulting in 837 bacterial reference genome clusters. Genomes with the greatest number of different marker proteins were selected as cluster-representatives. For every marker protein, alignments were built with MAFFT (v7.294b) [71] and subsequently trimmed with BMGE using BLOSUM30 [72]. Single protein alignments were then concatenated resulting in an alignment of 16,562 sites. Maximum likelihood phylogenies were inferred with IQ-TREE using 1000 bootstraps [73]. Trees were visualized with the R package ggtree [74].

Full-length 16S rRNA gene sequences were extracted from single-cell and population genomes with RNAmmer [75] and aligned with the SINA aligner [76]. Full-length sequences for the 16S rRNA gene phylogenetic tree were identified and extracted from ARB [77] from the SILVA SSU Ref NR 99 132 reference database [78]. A maximum likelihood tree was constructed using IQ-Tree with [73] 1000 bootstraps. Trees were visualized with ggtree [74].

### Phylogenetic placement of the Goldbacteria cellulase genes

To determine the nearest neighbors of the three cellulases from Goldbacteria with the highest expression, we constructed protein trees with all available hits from the public databases. Briefly, a Diamond blastp search was performed using the Goldbacteria MAGs and *Candidatus* ‘Cellulosimonas argentiregionis’ GBS−CoSAG_04. The top 100 hits for each protein were returned using an e-value cutoff of 1e-50. Redundancy was removed in the reference set by clustering with cd-hit at 99% sequence similarity. Sequences were then aligned (references and query sequences) with mafft-linsi [71], followed by trimming with trimal [79] at positions with less than 10% aligned information.

### Abundances of Goldbacteria across all IMG metagenomes

To estimate the relative abundances of members from the candidate phylum Goldbacteria in all publicly available assembled metagenomes in IMG/M [42], the full-length 16S rRNA gene sequence from the *Ca*.‘C. argentiregionis’ was BLAST searched against this set of metagenomic assemblies. Of the 6413 queried assembled metagenomes, only 46 samples contained contigs with hits of 85% or greater 16S rRNA gene sequence similarity over at least 500 bp range. The relative abundance of the identified metagenome 16S rRNA gene sequences was then estimated by comparing the read coverage of the 16S-containing contig against the read coverage for all other 16S-containing contigs within that sample. While hits from a range of environments were detected, the Obsidian Pool hot springs is the only sample site to contain >95% sequence similarity. Within this environment, relative abundances were well below 0.1%, and fell below 1% in nearly all other samples, suggesting this phylum has remained obscure within the rare biosphere. This same approach was applied to estimate diversity and abundance of the Elusimicrobia GBS-CoSAG_5. Environments with hits for the GBS-CoSAG_5 16S rRNA gene sequence were limited to hot springs environments and were only detected below 1%, strongly implicating this organism as an obligate thermophile of the rare biosphere. To calculate the read depth across all of the presented genome assemblies, reads from the metagenomes they originated from were mapped in an all vs. all analysis to our MAGs and SAGs representing Goldbacteria with bwa mem [80] and Samtools [81].

### Sequence data availability

All final genome data for this work can be found on the IMG website (https://img.jgi.doe.gov/). Co-assembled and final single-cell Genome IDs can be found in Supplementary Table 1. Metagenomes and the corresponding Genome IDs used to extract similar genomes to Goldbacteria are available in Supplementary Table 2. An OTU/relative abundance table for each of the three time points is available in Supplementary File 1. 16S sequences extracted from each of the genomes in Supplementary Table 1 are available in Supplementary File 2. Enzyme data with corresponding GenomeIDs and IMG_GeneIDs are available in Supplementary File 3. The original mini-metagenomes can also be collected from IMG using the Genome IDs present in Supplementary Table 4.

### Cell-free translation of putative cellulases and CBM genes

A set of 70 genes was identified from the 42 genomes with the potential for cellulose degradation (Supplementary File 3). Optimal gene lengths for protein translation were determined through iterative sequence alignment and comparison with crystal structures of homologs. Genes were synthesized at the Joint Genome Institute (Walnut Creek, CA) and cloned into the cell-free translation vector pEU [82]. Enzymes were expressed by sequential cell-free transcription-translation using wheat germ extract from Cell-Free Sciences (Yokohama, Japan) as described in detail in Takasuka et al. [83]. Briefly, plasmid DNA obtained from mini-prep was treated with proteinase K and further purified by extraction with phenol:chloroform. DNA sequences were transcribed to mRNA using SP6 polymerase, and mRNAs were mixed with wheat germ extract and translated in individual bilayered, diffusion-fed translation reactions. Product enzyme concentrations were measured by band analysis on stain-free SDS PAGE using unreacted wheat germ extract to correct the content of endogenous protein. Because wheat germ extract has no cellulase activity, translation reactions were used in catalytic assays without further purification.

### Functional validation of putative cellulases using oxime-NIMS

The translation reactions were diluted 4x in 50 mM sodium phosphate buffer pH 7.5; then 5 μL samples of the diluted translation reactions were transferred to a 96 well PCR plate. The PCR plate was sealed with Microseal B film (Bio Rad) and heated to the reaction temperature of 50 °C for 10 min and then cooled to 4 °C. Immediately after reaching 4 °C, 5 μL of 0.25 mM xylotetraose and 0.75 mM cellotetraose were added to each well. The plate was resealed and heated back to the original incubation temperature (50 °C) and samples were taken at 1, 4, and 20 h and stored at −20 °C. Oxime derivatization was performed by transferring a thawed 2 μL aliquot of the reaction mixture into a 96 well plate containing 8 μL of 100 mM glycine acetate, pH 1.4, 1.0 μL of an aqueous solution containing 2.5 mM of [U]-^13^C glucose and 2.5 mM of [U]-^13^C xylose, 2 μL of acetonitrile, 1 μL of methanol, 1 μL of NIMS oxime probe (10 mM in 1:1 (v/v) water:methanol), and 0.12 μL of aniline. The mixture was incubated at RT for 16 h before mass spectrometry-based analysis.

### Nanostructure-initiator mass spectrometry

The NIMS chips were produced as previously described [84]. The derivatization reaction samples were diluted 1:10 with 80/20 methanol/water containing 1% formic acid and then 6 μL of the mixture was transferred to a 384-well acoustic plate (Greiner Bio-one, Germany) for acoustic printing. The diluted derivatization reaction samples were acoustically printed onto an NIMS chip using EDC ATS-100 acoustic transfer system (BioSero, San Diego, CA) with a sample deposition volume of 10 nL. Samples were printed with the microarray spot pitch (center-to-center distance) set at 900 μm and samples were printed in duplicate. NIMS was performed using a 5800 MALDI TOF/TOF (AB/Sciex, Framingham, MA) mass spectrometer with a laser intensity of 4000 over a mass range of 500−2000 Da. The data collection was controlled using MALDI MSI 4800 imaging tool, each position on an NIMS chip accumulated 20 laser shots and scanning step size was set at 75 μm step both vertically and horizontally. Average signal intensities for the ions of the tagging products per sample spot were determined using the OpenMSI Arrayed Analysis Toolkit [85]. Negative control of nonenzymatic hydrolysis was subtracted to correct the calculated activities and the signal intensity of either [U]-^13^C glucose or [U]-^13^C xylose internal standards was used for normalization.

## Supplementary information

Supplementary Figures and Tables

16S itag data

16S rRNA gene sequences

Enzyme metadata

TAXON OIDS
