# Supplementary material for: Function-driven single-cell genomics uncovers cellulose-degrading bacteria from the rare biosphere
Source: ISME J. 2019 Nov 21;14(3):659–75. doi: 10.1038/s41396-019-0557-y (PMC7031533; doi:10.1038/s41396-019-0557-y)
Supplement: Supplementary file 1 — Supplementary Figures and Tables [file 41396_2019_557_MOESM1_ESM.docx]

**Supplementary Figures and Tables**


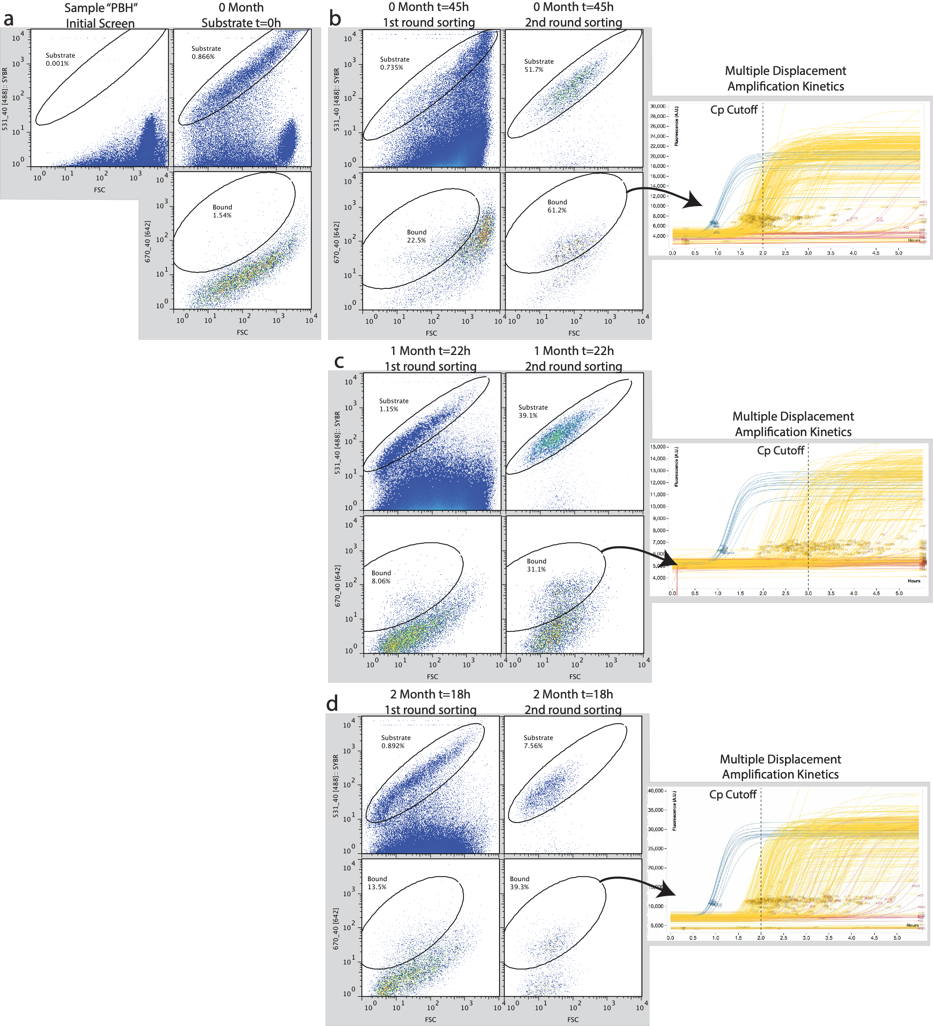


**Supplementary Figure 1**. Results of flow cytometry and cellulose-microbe particle whole genome amplification for GBS sample PBH at Months 0, 1, and 2. In each section (**a, b, c, d**) the Y axis of the upper flow cytometry dot plots [SYBR] represent green fluorescence used to identify labeled substrate. The Y-axis of the lower panels [670] represents red fluorescence used to identify when the cellulose particle is bound by a microbe. The X-axis [FSC] indicates forward scatter and relates to particle size. **a**) Initial screen of sample PBH to validate no overlap with fluorescently labeled substrate boundary, and screen immediately after addition of substrate where no microbe colonization is detected. The **b**), **c**), and **d**) panels display two-stage sorting for Month 0, 1, and 2, respectively. The MDA kinetic traces identify most strongly amplifying cellulose-microbe particles with lowest CP values, that were chosen for sequencing. Blue traces represent positive controls, yellow traces represent experimental cellulose-microbe particles, and red traces represent negative controls.


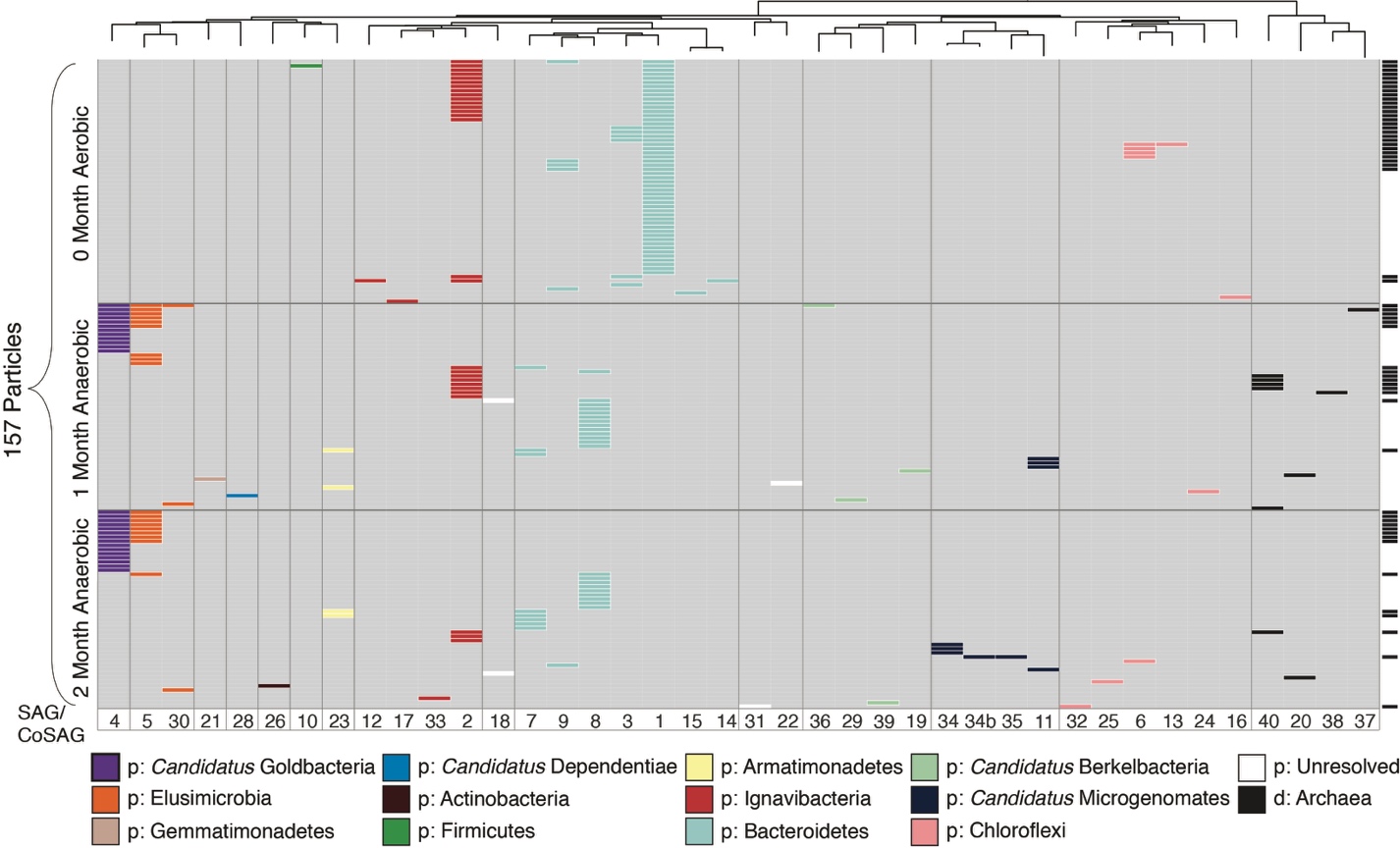


**Supplementary Figure 2**. Summary of all 157 sequenced cellulose-microbe particles. Each horizontal track within the grayed background region represents a sequenced particle, with each column representing a microbial cell found on that particle. The presence of a black hatch on the right side of the plot indicates that a particle contained more than one genome, i.e. the co-occurrence of more than one organism on a given particle. The dendrogram at the top of the plot represents the phylogenomic layout of the taxa extracted from the 157 particles.


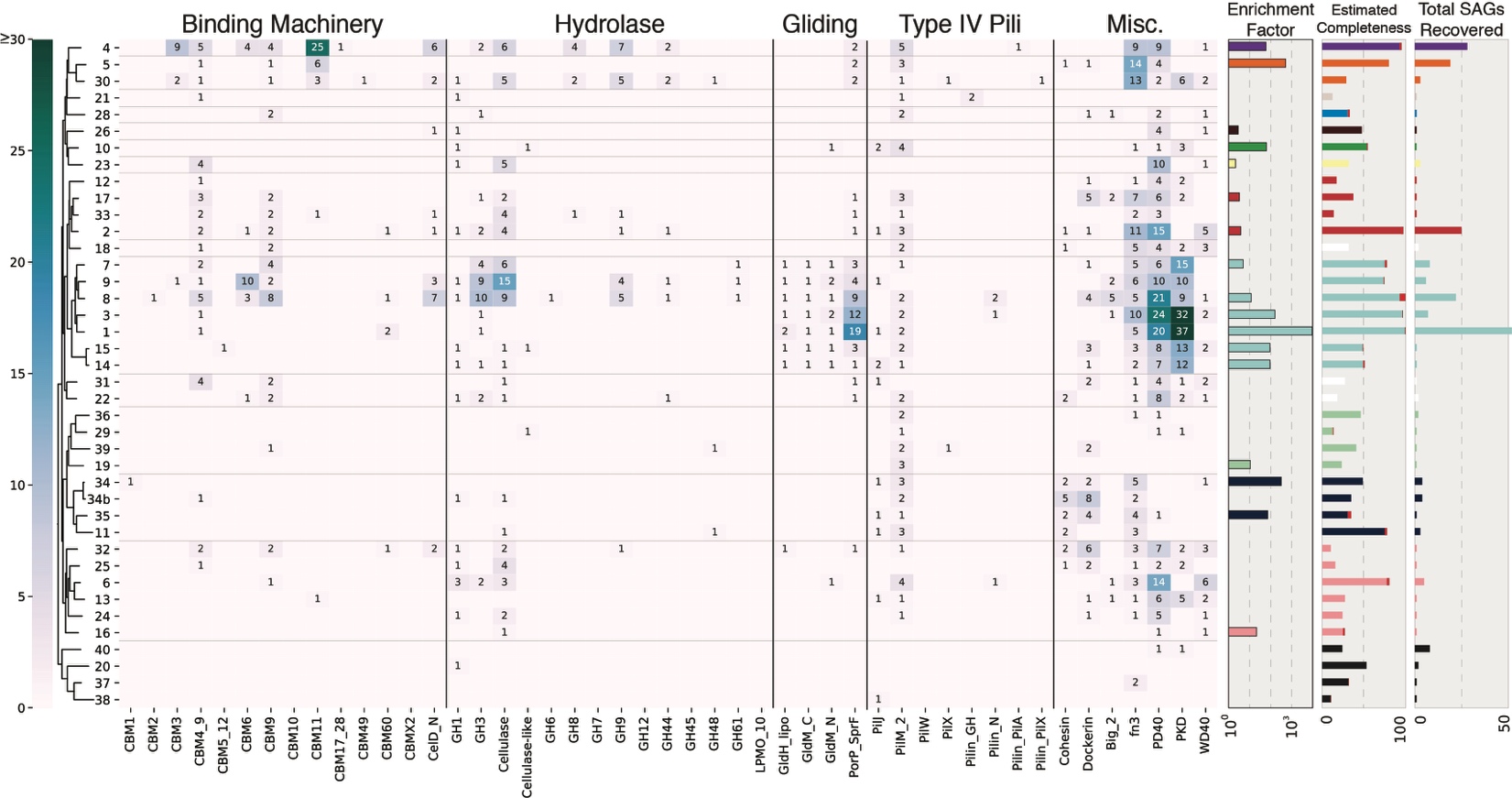


**Supplementary Figure 3**. Overview of all recovered microbial SAGs and CoSAGs from this function-driven cellulose screen. Heatmap presents an expanded profile of Pfams that potentially contribute to either adhesion or degradation of crystalline cellulose based on previous experimental validation. Bar graphs on the right represent the enrichment factor from the bulk pre-sorted population (when permitted by assembled 16S rRNA gene), genome completeness as estimated by CheckM, and total number of times each SAG was recovered. Graph colors correspond to phylogenetic colors in **Supplementary Figure 2**. A minimum of 50% estimated completeness is the required threshold to be considered a medium quality genome [29].


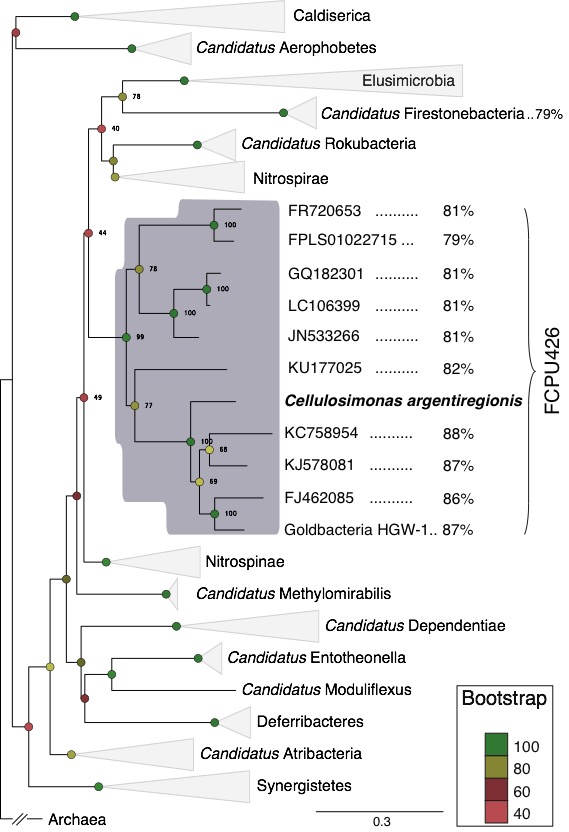


**Supplementary Figure 4**. 16S rRNA gene maximum likelihood tree placement of *Candidatus* ‘Cellulosimonas argentiregionis’ and Goldbacteria HGW-1 within the environmental FCPU426 candidate phylum group. Representative sequences were taken from the Silva database [78]. The grey box indicates the FCPU426 members.

**Supplementary Figure 5.** Bubble plot displaying the breadth of cellulose degrading machinery in Goldbacteria and other taxa identified using our function driven approach. Right-hand side plot displays the total counts of genes that may contribute to the degradation of cellulose and counts within each class. The unknown category includes beta-galactosidases and other glycoside hydrolases that could not be specifically linked to a cellulase.


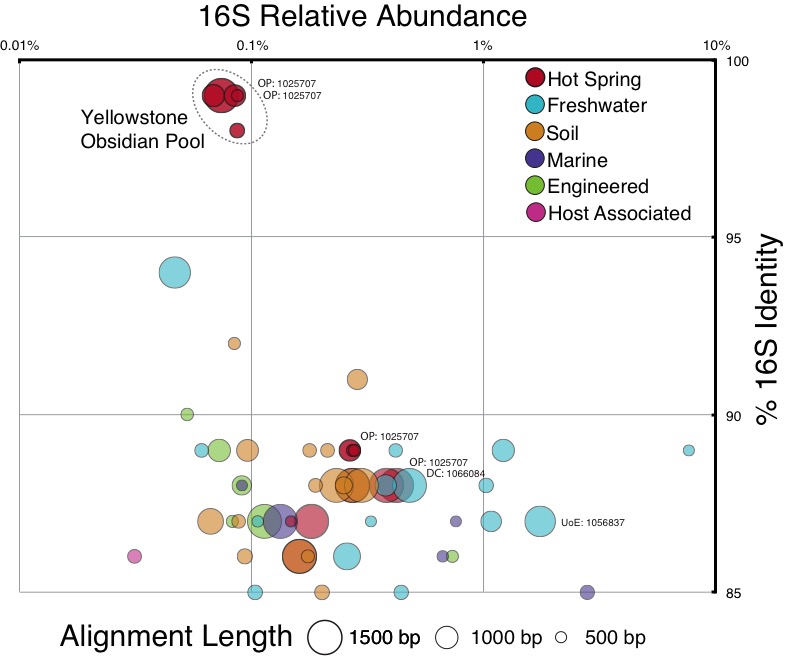


**Supplementary Figure 6**. 16S rRNA gene-based relative abundance of Goldbacteria detected in all assembled metagenomic samples in IMG/M (6,413 on Jan. 2018). Minimum blast hit requirements are 85% nucleotide identity over at least 500 bp.

**Supplementary Figure 7**. Read mapping depth of bulk reads from all publicly available metagenome samples (Hernsdorf et al. and JGI IMG/M) [41] containing Goldbacteria (X axis) to all representative draft genomes of members belonging to the candidate phylum Goldbacteria presented in Figure 4 (Y axis). No masking of conserved genome elements was performed. Black outlines highlight samples from which the corresponding MAG genome originated. Percent reads mapped to draft genome from each sample are displayed inside each box. Sample ID colors note biomes: Red = hotsprings, Green = engineered, Blue = freshwater.

**
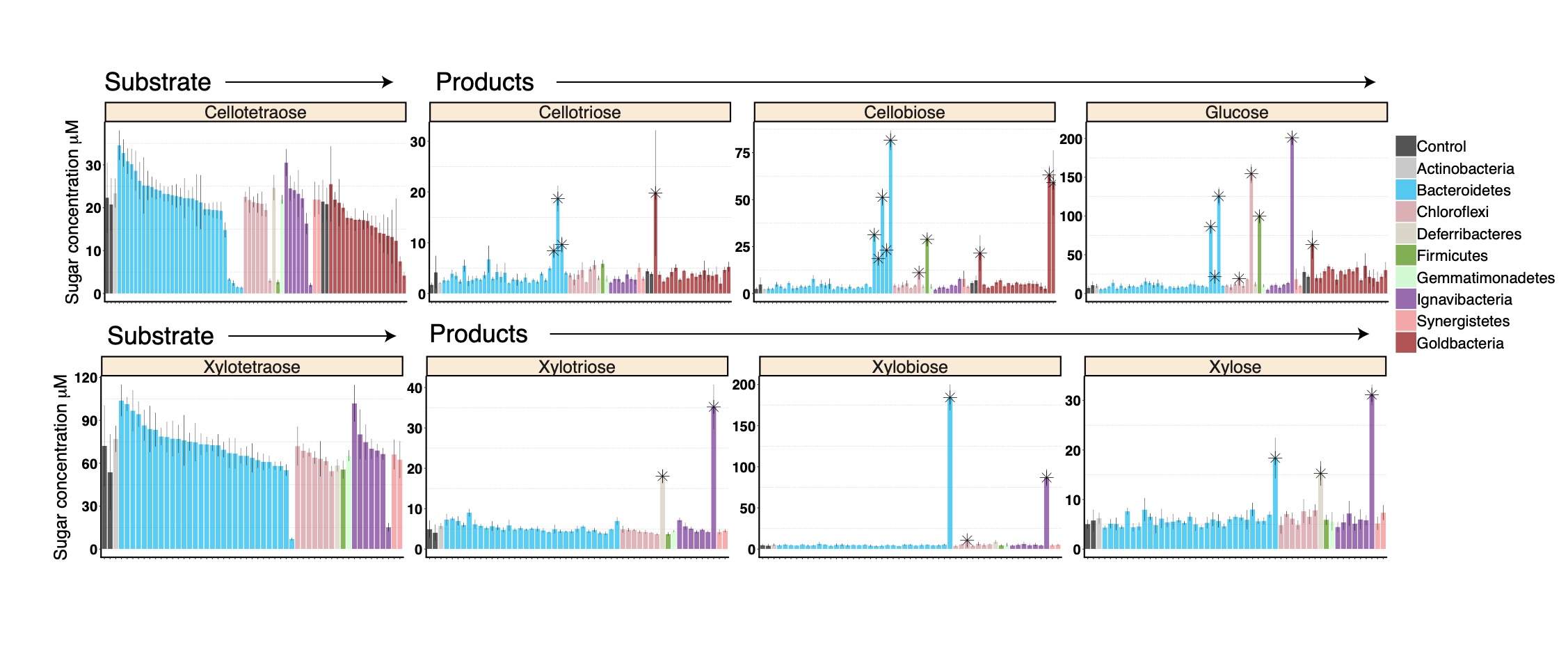
**

**Supplementary Figure 8**. Screening of the 70 potentially catalytic cellulase genes identified from the genomes of the Great Boiling Spring cellulose bound particles. The bar chart displays the initial substrate concentrations and the corresponding product concentrations. For an enzyme to be considered positive (denoted by the asterisk), the product concentration had to be at least 2X above the mean control concentrations. Buffer only and no gene controls are shown at left of each plot. Gene IDs and other associated metadata for each gene are provided in Supplementary_File_3_Enzyme_meta_data.txt where positive enzymes are noted as GH_active under the GH_activity column.

**
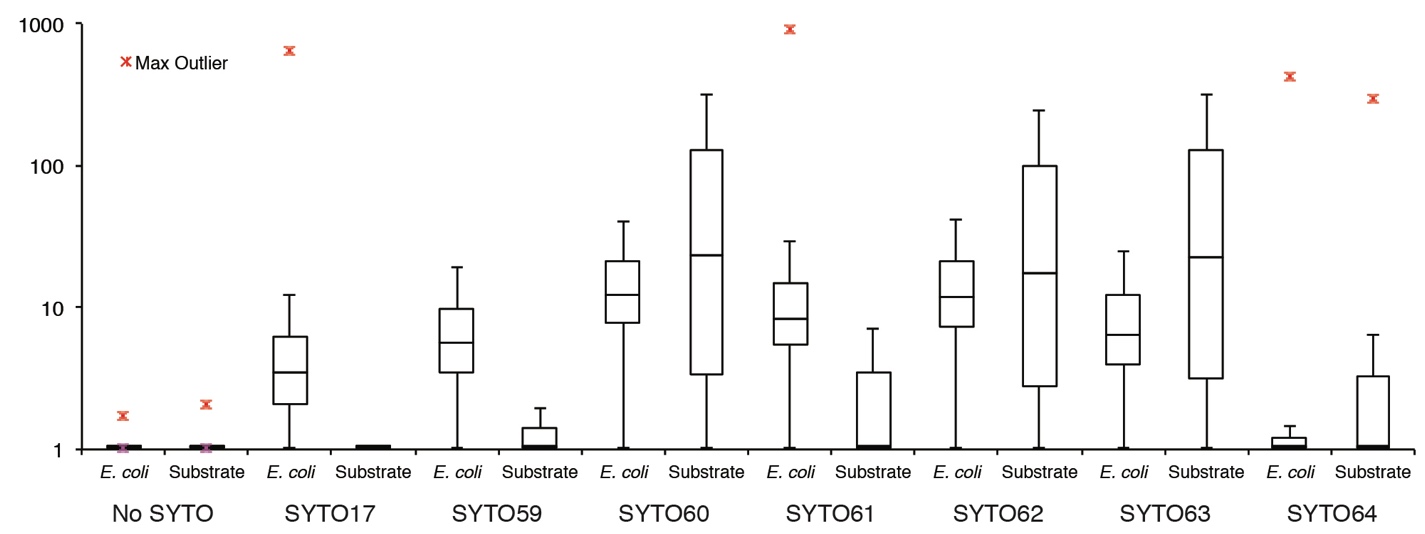
**

**Supplementary Figure 9**. Screening for compatible red fluorescent DNA stains. SYTO 17, 59 and 61 were chosen due to highest signal to noise ratio when measuring *E. coli* vs. non-specific association of the dye with the cellulose substrate. SYTO 60, 62, 63, and 64 seem to impart a large non-specific red fluorescence on the abiotic cellulose particles, making resolving cellulose-microbe particles more difficult.

**Supplementary Table 1:** Genomes from GBS with associated metadata including genome quality in the form of CheckM completion/contamination estimates, Genome size estimated by CheckM, and the fold enrichment in iTag 16S rRNA gene amplicon data corresponding to incubations at Month 0, Month 1 and Month 2. The phylum assignments are taken from the marker gene genome tree (see **Figure 3**). IMG Genome IDs are provided for reference. Row numbers refer to the genome IDs plotted in Supplementary Figures 2 and 3.

**Supplementary Table 2:** IMG/M samples where Goldbacteria MAGs were recovered.

**Supplementary Table 3:** Environmental sample properties and isolates used.
